# Exploring Transfeminine Youth Health Disparities in Thailand: An Online Survey Analysis of Characteristics and Hormonal Use Patterns

**DOI:** 10.1089/heq.2023.0258

**Published:** 2024-09-26

**Authors:** Nadvadee Aungkawattanapong, Thitaporn Prownpuntu, Chansuda Bongsebandhu-phubhakdi

**Affiliations:** ^1^ Department of Pediatrics, Faculty of Medicine, Chulalongkorn University and King Chulalongkorn Memorial Hospital, Bangkok, Thailand.; ^2^ Center of Excellence in Transgender Health (CETH), Chulalongkorn University.

**Keywords:** transgender, youth, Thai, transfeminine

## Abstract

**Background:**

Transgender youth in Thailand often encounter limitations when accessing gender services, leading many to use nonprescribed hormones.

**Objectives:**

This study aimed to explore 1) the pattern of Gender-Affirming Hormone Treatment (GAHT) use among Thai transfeminine youth, 2) the baseline characteristics of transfeminine youth, and 3) the self-reported happiness score and depression screening.

**Methods:**

We conducted a cross-sectional online survey among Thai transfeminine youth aged 12 to 25 years. Data were collected using a self-administered questionnaire distributed via social media platforms from September 2021 to October 2022.

**Results:**

Of the 256 participants, 226 (88%) reported having used hormones. The average age at which participants first used hormones was 15.5 years (SD 2.5). A majority (94.6%) of those who had used hormones disclosed their gender identity, compared with a lower percentage (60%) in the nonhormone use group. Among those with hormone use experience, only 36.1% reported use that strictly aligned with the recommended regimen, according to the 2017 Endocrine Society Clinical Practice Guideline, which includes an antiandrogen agent (oral cyproterone acetate) and estrogen (either oral estradiol valerate, oral 17 beta-estradiol, or estrogen gel). Furthermore, the average happiness scores and PHQ-A scores showed no significant differences between individuals who have or have not used GAHT.

**Conclusion:**

Thai transfeminine youth have started using GAHT during adolescence. However, many of them use it in ways that deviate from the recommended standard of gender care. The findings underscore the urgent need to enhance medical access, education, and supervision for gender health care among transfeminine youth.

## Introduction

Transfeminine individuals, a subgroup within the spectrum of transgender and gender diversity, are those assigned male at birth but identify as women, regardless of whether they have undergone any form of gender transition.^1^ Some transgender people experience gender dysphoria, which involves distress due to a mismatch between one’s gender identity and assigned sex. Recent studies have advised against gender conversion therapies, which attempt to change an individual’s gender identity to align with their sex assigned at birth^2,3^

In line with the World Professional Association for Transgender Health (WPATH) Standards of Care and the 2017 Endocrine Society Clinical Practice Guideline,^1,4^ the Thai guideline for gender-affirming care for youth includes initial thorough assessments of gender dysphoria to determine the appropriateness of starting gender-affirming hormone therapy (GAHT). The GAHT protocol involves antiandrogen agents such as oral cyproterone acetate or spironolactone, alongside estradiol administered as oral estradiol valerate, oral 17 beta-estradiol, or estrogen gel. Providers also address associated mental health issues and medical complications, advocating a holistic approach.^5^ However, transgender individuals in many developing countries, including Thailand, can obtain oral contraceptive pills (OCPs) over-the-counter and access hormones and other medications through unauthorized sellers. A 2013 study also highlighted that transgender individuals in Thailand predominantly rely on hormone therapy recommendations from friends or acquaintances.^6,7^

Despite established guidelines, numerous health disparities hinder transgender youth’s access to health care in Thailand. These include limited access to knowledgeable providers, discrimination in medical settings, financial barriers to treatments, high costs of services, and parental consent requirements for minors.^8,9^ Although adults have broader access to gender health services in major urban centers, options for adolescents and young adults are mainly confined to certain university hospitals in Bangkok. In 2023, efforts to enhance youth access led to the establishment of new gender health care facilities in medical schools across urban centers such as Chiang Mai, Khon Kaen, and Songkhla. Nonetheless, the centralized nature of these services poses significant challenges for transfeminine youth seeking gender-affirming care. Furthermore, despite universal public health coverage in Thailand since 2002 through the Universal Coverage Scheme, the Civil Servant Medical Benefit Scheme, and the Social Health Insurance Scheme, none include gender-affirming care provisions. As a result, all expenses for laboratory tests and medications are out-of-pocket, limiting access to necessary evaluations, hormone risk assessments, and follow-up protocols for transfeminine individuals.^6,7,10^

Limited data exist on the characteristics and patterns of hormonal use among transfeminine youth, especially in the Thai context. Therefore, this study aims to explore the patterns of GAHT use among transfeminine youth, examine their baseline characteristics, and evaluate the relationship between the use of gender-affirming hormones, self-reported happiness, and depression screening results using the Patient Health Questionnaire-A (PHQ-A).

## Methods

The research used a cross-sectional methodology and was conducted between September 2021 and October 2022. The survey was administered to individuals between the ages of 12 and 25 who self-identified as transfeminine. The questionnaire was developed specifically for this study. The survey questions were designed to align with the study’s objectives. A pilot study involving 20 participants was conducted to ensure the clarity of the questions and the practicality of the self-administered questionnaire method. No revisions were necessary.

The recruitment for the study was conducted online through multiple social media platforms connected to our network of gender health clinics, such as an official LINE account, Facebook, and Twitter. In addition, participants were asked to share information about the research study with their transfeminine friends and within relevant social media groups. Participants responded to the survey using Google Forms. The confidentiality of the results was strictly maintained, and only authorized personnel had access to them. All participants were required to respond to the questionnaire themselves and individuals with cognitive and/or vision disabilities were excluded from the study. Ethical approval for the study was obtained from the Institutional Review Board of the Chulalongkorn University Faculty of Medicine in Bangkok, Thailand (No. 613/64).

When participants accessed the online survey, the first page presented an information sheet about the research study. This sheet included the purpose of the study, the research methodology, and all relevant details for participants. If participants voluntarily chose to participate in the study, they would tick the box indicating their understanding and their willingness to participate in the research study. Parental consent was waived for participants aged 12–18 years due to the minimal risk and potential harm associated with disclosing their LGBTQ identity to their parents. To ensure confidentiality and privacy, no personal details or names were collected.

The survey was structured into four sections. The primary section aimed to validate the accuracy of the participants’ assigned sex at birth and their self-identified gender. The subsequent segment focused on gathering sociodemographic information pertinent to the study population. The third part inquired about the disclosure of their gender identity and their self-reported happiness score. Finally, the fourth section explored the experiences with the use of sex hormones and/or other relevant medications.

To determine the gender identity of the participants, this study used specific questioning strategies. The initial inquiry asked participants, “What is your gender identity?” with dichotomous choice of transfemine individual (in Thai language: kathoey, phuying kham phet, and sao praphet song) or not. This question was reiterated in the sociodemographic section. A subsequent question aimed to further explore gender identity, specifically addressing individuals who identify as transfeminine person. The question posed was as follows: “Some individuals describe themselves as transfeminine when they were assigned a male at birth but identify with a female gender identity in terms of their thoughts and feelings. Do you identify yourself as transfeminine (in Thai language: kathoey, phuying kham phet, and sao praphet song)?” The participants were presented with four response options: A. No, I do not identify as transfeminine person, B. Yes, I identify as a transfeminine person, C. I am uncertain about my identification as transfeminine person, and D. I do not understand the meaning of this question.^11^ Data were only retained for individuals who specifically indicated their response as B.

The subsequent section of the survey encompassed a variety of inquiries related to various demographic and health-related factors. Participants were asked to provide information on their gender, age, presence of concurrent medical and psychiatric illness, characteristics of their religious affiliation, smoking habits, alcohol consumption, exercise frequency (more or less than three times per week), and use of gender-affirming hormones. The use of gender-affirming hormones, particularly antiandrogen and estrogen in this study, was classified into two groups: those who had used such hormones and those who had never used them. Participants reporting having used hormones were asked to provide the specific names of the hormones/drugs they had used.

The presentation of sociodemographic and anthropometric data involved reporting the mean with standard deviation (SD) or the median with the interquartile range for continuous variables. Categorical variables were displayed using frequencies and percentages. Group comparisons were conducted using an independent sample T-test, with statistical significance set at a *p* value <0.05. Data analysis was performed using IBM SPSS Statistics for Windows, Version 28.0. Armonk, NY: IBM Corp.

## Results

Among those who reported hormone use, the mean age was slightly higher at 20.0 years (SD = 3.1), while those who never used hormones had a lower mean age of 18.0 years (SD = 3.1) (*p* = 0.001). The mean age at the hormone use initiation was 15.5 years (SD = 2.5). This indicates that participants generally initiated hormone uses during their midadolescence. Of those hormone users, only 39 percent reported using it regularly. Other demographic details can be found in Table 1.

**Table 1.** Demographic Characteristics of the Study Participants

	Total	Ever use hormone	Never use hormone	
	*n* = 256	*n* = 226	*n* = 30	*p* value
Age, mean (SD)^*^	19.7 (3.2)	20.0 (3.1)	18.0 (3.1)	0.001
Age at first hormone use (years), mean (SD)		15.5 (2.5)		
Height (cm), mean (SD)	170.4 (6.8)	170.6 (6.2)	169.0 (10.3)	0.595
Weight (kg), mean (SD)	61.1 (14.9)	60.9 (14.5)	62.8 (17.8)	0.589
BMI (kg/m^2^), mean (SD)	21.0 (4.6)	20.8 (4.5)	21.8 (4.8)	0.111
Religion				0.529
Buddhism, number (%)	222 (86.7)	193 (85.4)	29 (96.7)	
Christianity, number (%)	7 (2.7)	7 (3.1)	1 (3.3)	
Islam, number (%)	8 (3.1)	8 (3.5)	0 (0)	
Hinduism, number (%)	1 (0.4)	1 (0.4)	0 (0)	
no religion, number (%)	18 (7)	18 (7.5)	0 (0)	
Smoking				0.402
Never, number (%)	230 (89.8)	201 (88.9)	29 (96.7)	
Irregular smoking, number (%)	21 (8.2)	20 (8.8)	1 (3.3)	
Regular smoking, number (%)	5 (2.0)	5 (2.2)	0 (0)	
Alcohol consumption				0.519
Never, number (%)	113 (44.1)	98 (43.4)	15 (50)	
Irregular drinking, number (%)	139 (54.3)	125 (55.3)	14 (46.7)	
Regular drinking, number (%)	4 (1.5)	3 (1.3)	1 (3.3)	
Physical exercise				0.330
Not usual, number (%)	74 (28.9)	64 (28.3)	10 (33.3)	
Sometime, number (%)	153 (59.8)	134 (59.3)	19 (63.3)	
Regular exercise, number (%)	28 (11.3)	28 (12.4)	1 (3.3)	
Disclosure of their gender identity (%)	232 (90.6)	214 (94.7%)	18 (60%)	0.947

^*^ *p* < 0.05, (Independent sample *t-test*).

The types of GAHT are reported in Table 2. Oral cyproterone acetate was widely used, with 139 participants (52.3%) reporting its use. The most commonly used hormone was oral estradiol valerate, reported by 123 participants (46.2%). Oral 17 beta-estradiol was the second most frequently used hormone, with 102 participants (38.4%) reported use. In addition, ten participants (3.8%) have used estrogen gel. There were 65 participants (28.7%) who reported using various brands of oral contraceptive pills, most of which contained cyproterone acetate and ethinyl estradiol, for gender transition. Furthermore, a smaller proportion of participants reported using other methods, including herbs (20 participants, 7.5%), estrogen injections (6 participants, 2.3%), and hydroxyprogesterone caproate injections (4 participants, 1.5%).

**Table 2.** Types of Gender Affirming Hormone/Medication Used by Thai Transfeminine Youth

Types of hormone/medication	*n*	%
Oral cyproterone acetate	139	52.3
Oral estradiol valerate	123	46.2
Oral estradiol	102	38.4
Oral contraceptive pills	65	28.7
Oral phytoestrogen	20	7.5
Estrogen gel	10	3.8
Estrogen/estradiol injection	6	2.3
Hydroxyprogesterone caproate injection	4	1.5

**FIG. 1.**
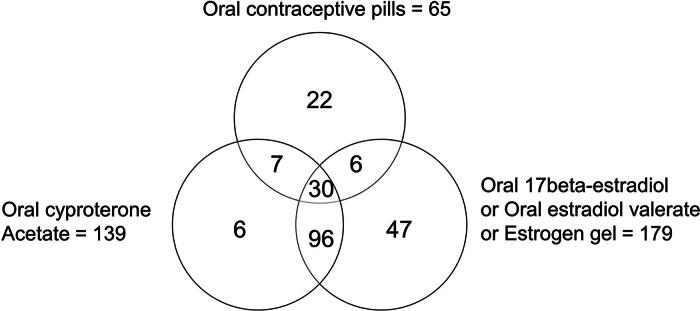
Venn Diagram of Types of Gender Affirming Hormone/Medication Used by Thai Transfeminine youth.

The Venn diagram in Figure 1 illustrates the overlapping data of the drug/hormones used by the participants. Approximately a third of the participants (96 participants, 44.9%) reported using cyproterone acetate with estrogen (oral estradiol valerate, oral 17 beta-estradiol, or estrogen gel), which aligns with recommended hormonal therapy guideline by WPATH and the Endocrine Society.^1,4^ However, the remaining participants used the drug/hormones in ways that deviated from the recommended guidelines. Among the hormone users, 133 participants (58.8%) reported using androgen blocker (cyproterone acetate) with other forms of estrogen, while 75 individuals (33.2%) reported using estrogen alone. In addition, there are 36 participants (15.9%) who reported using both estradiol and oral contraceptive pills interchangeably. Regarding their lifetime hormonal therapy exposure, 30 participants (13.3%) of hormone users reported having used more than one hormonal treatment regimens.

Most of the participants (86.7%) identified themselves as Buddhists, while Islam and Christianity were represented by 3.1% and 2.7% of the participants, respectively. A notable proportion of the sample (7%) reported having no religious affiliation and a participant identified as Hindu, representing 0.4% of the sample.

Looking into other adolescent health-related issues, we found that there is no significant difference in growth parameters between hormone users and nonhormone users. The anthropometric measurements of height, weight, and body mass index (BMI) were compared between the groups of participants who had used hormones and those who had never used hormones. No significant differences were found. The mean height of all participants was 170.4 centimeters (SD 6.8). Similarly, the mean weight was 61.1 kg (SD 14.9). The calculated mean BMI was 21.0 kg/m2 (SD 4.6).

Regarding other adolescents’ health-related behaviors, we found that 89.8% of the total sample had never smoked and 44.1% of the participants reported never drinking alcohol. No statistically significant differences were found in smoking and alcohol consumption between participants who had used gender affirming hormones and those who had never used them. In terms of physical exercise, majority of the overall participants (59.8%) reported exercising occasionally (less than 3 times per week). Among hormone users, 134 participants (59.3%) who had used hormones reported exercising occasionally, while 63.3% of nonhormone user reported the same frequency of exercise.

In terms of social transitioning and gender identity disclosure, 90.6% of the participants reported disclosing their gender identity to others. Among hormone users, a slightly higher percentage of 94.6% revealed their gender identity, while those who never used hormones, only 60% have disclose their gender identity to others. Statistical analysis using a chi-square test indicated that there were no significant differences in disclosure rates between hormone users and nonusers (*p* = 0.947).

For mental health screening, we used PHQ-A screening tool and self-reported happiness scores, a scale ranging from 0 to 10, with higher scores indicating higher levels of happiness. The overall mean of PHQ-A score was 9.2 (SD = 6.5). There is a slight difference in mean of PHQ-A score among hormone users and nonhormone user, 9.3 (SD = 6.1) and 7.8 (SD = 7.3), respectively. However, the difference is not statistically significant (*p* = 0.220). The same trend is found using self-report happiness scale. The mean happiness score for the total sample was 7.1 (SD = 2.0). Among hormone users, the mean happiness score was 7.0 (SD = 2.0), and only a slightly higher score among those who never used hormone at 7.3 (SD = 2.0) without statistical significance (*p* = 0.389).

## Discussion

This study investigates the characteristics and patterns of gender-affirming hormone usage among transfeminine youth in Thailand, considering their religious and cultural backgrounds. Baseline characteristics, including age, height, weight, BMI, and health-related behaviors such as smoking, alcohol consumption, and physical exercise, showed no significant differences between hormone users and nonusers. The religious affiliations recorded—Buddhism (92.5%), Islam (5.4%), and Christianity (1.2%)—correspond with the national data from 2021.^12^

Most participants had disclosed their gender identity and undergone social transitions, irrespective of hormone therapy usage. The openness of Thai society may help alleviate the stress associated with these transitions compared with many countries.^6,13,14^

Among the 256 participants, a significant majority (88.2%) reported using gender-affirming hormones, higher than the GAHT usage rates reported in previous adult studies in Thailand (64.6–81%).^7,15^ It should be noted that this study uniquely focuses on the experiences of young Thai transfeminine youth and their lifetime exposure to GAHT. The use of an internet-based platform for recruitment might have attracted a cohort particularly interested in GAHT, potentially skewing the reported usage rates.

In our study, the average age at which transfeminine individuals began GAHT was 15.5 years (SD 2.5). This initiation age is notably later than that reported in earlier adult studies, which indicated an average initiation age of approximately 10 years.^6,16^ Notably, only 39% of participants used GAHT regularly, a concern given the potential for irregular hormone use to result in serum hormone concentrations outside the optimal range. Supraphysiological hormone concentrations can increase the risk of adverse events such as venous thrombosis, cholelithiasis, transaminitis, and hypertriglyceridemia. Conversely, discontinuing hormone therapy or maintaining suboptimal hormone levels can lead to an increased risk of accelerated bone loss.^4,17^

During our study period, gender-affirming services were limited, but this was not the only barrier encountered by transfeminine youth. Other factors, such as lack of family acceptance, and unsafe school environments also hindered access to gender care.^8,18,19^ This study found that many transfeminine youth resort to using nonrecommended medications and OCPs, underscoring significant misconceptions and knowledge gaps about GAHT. In addition, the financial challenges are profound, as government hospitals do not cover gender care-related expenses, affecting particularly those without personal income or caregiver support. This often forces them to seek hormones from unregulated sources or opt for cheaper, over-the-counter alternatives like OCPs.^11,13^

The study revealed no significant differences in depression screening or self-reported happiness between those who had used GAHT and those who had not, suggesting that happiness and mental health are influenced by a broader range of life factors such as relationships, support systems, and social status. Evaluating the psychological well-being of transfeminine youth warrants further longitudinal cohort studies in the future.

Finally, while this study provides valuable insights into the use of unprescribed GAHT among transfeminine youth in Thailand and underscores the need for better educational efforts on GAHT use and health monitoring, it faces limitations such as potential selection bias from recruiting via a gender health care center’s social media platform and the inherent issues with self-reported data. Future research should emphasize qualitative studies to further explore the attitudes and needs of transfeminine youth, aiming to enhance access to and the quality of gender-affirming treatments. This approach will highlight the urgent need for tailored health care interventions to support this vulnerable population.

## Conclusion

This study has highlighted key aspects of gender-affirming hormone use among transfeminine youth in Thailand, revealing both high rates of usage and significant irregularities in administration. The data suggest that societal openness in Thailand may mitigate some transitional stresses, yet substantial barriers remain, notably in health care affordability and access. The irregular use of prescribed hormones, often supplemented by unregulated sources or inappropriate alternatives such as OCPs, underscores the urgent need for comprehensive health care support and education. The findings emphasize the importance of enhancing health care services and public awareness to reduce health disparities among transfeminine youth and ensure safer, more effective gender-affirming treatments. Targeted interventions and sustained research are crucial for ensuring safer, more effective treatments and enhancing their overall well-being in Thailand.

## Data Availability

The datasets used and/or analyzed during the current study are available from the corresponding author on reasonable request.

## Abbreviations Used

GAHT: Gender-affirming hormone treatment
OCPs: Oral contraceptive pills
PHQ-A: Patient health questionnaire-A
